# Flavonoids uptake and their effect on cell cycle of human colon adenocarcinoma cells (Caco2)

**DOI:** 10.1038/sj.bjc.6600295

**Published:** 2002-05-03

**Authors:** M Salucci, L A Stivala, G Maiani, R Bugianesi, V Vannini

**Affiliations:** National Institute of Food and Nutrition Research, Via Ardeatina 546, 00178, Rome, Italy; Department of Experimental Medicine, Unit of General Pathology, University of Pavia, P.za Botta 10, 27100, Pavia, Italy

**Keywords:** Caco2, flavonoids, antioxidant activity, cell cycle

## Abstract

Green tea, mainly through its constituents epigallocatechin gallate, epigallocatechin, epicatechin gallate and epicatechin, has demonstrated anticarcinogenic activity in several animal models, including those for skin, lung and gastro-intestinal tract cancer, although less is known about colorectal cancer. Quercetin, the major flavonoid present in vegetables and fruit, exerts potential anticarcinogenic effects in animal models and cell cultures, but less is known about quercetin glucosides. The objectives of this study were to investigate (i) the antioxidant activity of the phenolic compounds epicatechin, epigallocatechin gallate, gallic acid and quercetin-3-glucoside; (ii) the cytotoxicity of different concentrations of epicatechin, epigallocatechin gallate, and gallic acid; (iii) the cellular uptake of epicatechin, epigallocatechin gallate, gallic acid and quercetin-3-glucoside and (iv) their effect on the cell cycle. Human colon adenocarcinoma cells were used as experimental model. The results of this study indicate that all dietary flavonoids studied (epicatechin, epigallocatechin gallate, gallic acid and quercetin-3-glucoside) show a significant antioxidant effect in a chemical model system, but only epigallocatechin gallate or gallic acid are able to interfere with the cell cycle in Caco2 cell lines. These data suggest that the antioxidant activity of flavonoids is not related to the inhibition of cellular growth. From a structural point of view, the galloyl moiety appears to be required for both the antioxidant and the antiproliferative effects.

*British Journal of Cancer* (2002) **86**, 1645–1651. DOI: 10.1038/sj/bjc/6600295 www.bjcancer.com

© 2002 Cancer Research UK

## 

Epidemiological studies from around the world have consistently reported that a high intake of fruit and vegetables is associated with a low incidence of most types of cancer (Wang *et al*, 1989; Dragsted *et al*, 1993). It is estimated that about 70% of all cancers are attributable to the diet. Colorectal cancer is the second most prevalent cancer in the developed world (Parkin *et al*, 1985). Western diet with a high intake of calories from fat and low fibre supply has been linked to an increase of colon cancer incidence, whilst vegetarian or mediterranean diets are more protective (Vargas and Alberts, 1992). Several compounds have been identified in plants which have well recognised antioxidant properties, such as carotenoids, ascorbic acid, α-tocopherol and flavonoids (Block *et al*, 1992; Hertog *et al*, 1992) and inhibit cancer development.

Thus, the use of natural substances, that are derived from the diet for chemoprevention, might provide a strategy to inhibit the development of cancer. Green tea, mainly through its constituents epigallocatechin gallate (EGCG), epigallocatechin (EGC), epicatechin gallate (ECG) and epicatechin (EC), has demonstrated anticarcinogenic activity in several animal models, including those for skin, lung and gastro-intestinal tract cancer (Katiyar *et al*, 1992; Yang and Wang, 1993). Several recent studies (Berger *et al*, 2001; Uesato *et al*, 2001) on the effect of green tea polyphenolics on colon cancer cell lines have shown antiproliferative activity. Controversial results have been obtained by other authors on animal model (Hirose *et al*, 2001) and on human model (Nagano *et al*, 2001). *In vitro* studies (Komori *et al*, 1993) have shown that green tea can arrest the growth of human mammary and lung cancer cell lines. New data (Cao *et al*, 1999) indicate that EGCG suppresses endothelial cell growth *in vitro* and the formation of new blood vessels in chick chorioallantoic membrane; drinking green tea significantly prevents corneal vascularisation induced by one of the most potent angiogenetic factors (VEGF). The same results have been obtained by Jung *et al* (2001) in *in vitro* studies, in serum-deprived HT29 cells, and in *in vivo* studies, on the growth of HT29 cells in nude mice. EGCG exerts, at least part of its anticancer effect, by inhibiting angiogenesis through blocking the induction of VEGF in both *in vitro* and *in vivo* studies, while EGC, ECG and EC have not demonstrated the same effect (Jung *et al*, 2001). However, green tea extract has a stronger effect on cell growth than the major phenolic component, EGCG (Komori *et al*, 1993). Recently, another study (Suganuma *et al*, 1999) has demonstrated a synergic effect of EGCG and EC in the cell line PC-9, inhibiting growth.

Quercetin and its glucosides are the major flavonoids present in vegetables, in particular fresh onions, fruit (Hertog *et al*, 1992; 1993) and beverages (Kiviranta *et al*, 1988). Animal (Deschner *et al*, 1991; Yang *et al*, 2000) and *in vitro* studies suggest that quercetin exerts a preventive effect against cancer (Kawaii *et al*, 1999), but less is known about quercetin glucosides. Hollman *et al* (1995) show that 52% of the quercetin glucosides from onions was absorbed, whereas only 24% of free quercetin was absorbed in human model. It would therefore appear that the quercetin glucosides are better absorbed than the free aglycone. However, the specific role of individual phenols in carcinogenesis has been and still is very unclear due to controversial results.

The objectives of this study were to investigate (i) the antioxidant activity of the phenolic compounds EC, EGCG, gallic acid (GA) and quercetin-3-glucoside (IQ); (ii) the cytotoxicity of different concentrations of EC, EGCG, and GA; (iii) the cellular uptake of EC, EGCG, GA and IQ; and (iv) their effect on the cell cycle. Human colon adenocarcinoma (Caco2) cells were used as experimental model. These cells, chosen because of their great capacity to differentiate at confluence and to display specialised enterocyte/colonocyte cell function, express many intestinal enzymes such as sucrase, maltase, gammaglutamyltransferase and aldehyde deydrogenase comparable with normal colonic mucosal activity (Koivisto and Salaspuro, 1997) and retain the ability to transport vitamins, ions and glucose (Pinto *et al*, 1983).

## MATERIALS AND METHODS

### Chemicals

Nonidet P-40, propidium iodide (PI), RNase A, bromodeossiuridine (BrdU), Tween 20, tris-HCl, MgCl_2_, HCl, L-glutamine (2 mM), streptomycin, penicillin, amphotericin B (fungizone), R-phycoerythrin (R-PE), bovine serum albumin (BSA), fluoresceinisothiocyanate (FITC)-conjugated anti-mouse antibody, sodium tetraborate, and phosphate buffer solution (PBS) were purchased from Sigma (Milan, Italy). Anti-BrdU monoclonal antibody was purchased from Dako (Denmark). Phenylmethylsulphonylfluoride (PMSF) and propidium iodide (PI) were purchased from BDH (Poole, UK). Foetal calf serum (FCS) was purchased from Gibco (UK). Dulbecco Modified Medium (D-MEM) was obtained from Bio-Whittaker (UK). Ammoniumiron(II)sulphatehexahydrate, xylenol orange sodium salt and hydrogen peroxide (H_2_O_2_) were purchased from Aldrich (Milan, Italy). 2,2′-diazobis(2-amidinopropane) dihydrochloride (ABAP) was purchased from Wako (Società Italiana Chimici, Rome, Italy). EGCG, EC and GA were purchased from Sigma (Milan, Italy) while IQ was purchased from Extrasynthese (Societá Italiana Chimici, Rome, Italy). Ethanol and methanol were purchased from Carlo Erba (Milan, Italy). EGCG, EC, GA and IQ were dissolved in methanol. The final methanol concentration in the cell incubation medium was 0.1%.

### Measurement of the antioxidant activity

The antioxidant activity of EGCG, EC, GA and IQ was evaluated *in vitro* by measuring the total radical antioxidant potential (TRAP) according to Ghiselli *et al* (1995). This method is based on the protection afforded by the antioxidants present in the sample against the decay of a fluorescent target R-phycoerythrin (R-PE) during a controlled peroxidation reaction induced by the free radical generator 2,2′-diazobis(2-amidinopropane) dihydrochloride at 37°C.

### Analysis of hydrogen peroxide

In each sample (DMEM with and without 100 μM of EGCG, EC, GA and 70 μM IQ) the formation of hydrogen peroxide (H_2_O_2_) was assayed using the FOX2 reagent (ferrous in xylenol orange, version 2) according to the procedure of Nourooz-Zadeh *et al* (1994). One hundred μl of sample was mixed with 900 μl of FOX2 reagent, incubated at room temperature for 30 min in 1.5 ml microcentrifuge vials, then centrifuged at 12 000 **g** for 5 min. Absorbance of the supernatant was then read at 560 nm.

### Cell culture

The human colon adenocarcinoma Caco2 cells were purchased from American type culture collection (Rockville, USA). Cells were cultured in D-MEM, supplemented with 15% of FCS, L-glutamine (2 mM), streptomycin and penicillin (100 μg/100 U ml^−1^) and 2.5 μg ml^−1^ fungizone, and incubated at 37°C in a 5% CO_2_ incubator. Cells were plated at a density of about 1×10^6^ cells/flask and grown until reaching confluence.

### Cytotoxicity

When the cells had reached the 60–70% of confluence, the growth medium was replaced with a medium containing 15% of FCS and various concentrations of EGCG, EC, and GA (100, 125, 250, 500 μM). Incubation with these phenolic compounds was performed for 24 h. The toxicity of IQ was analysed up to the limit of solubility (70 μM) and it was not toxic for the cellular line (data not shown). After the treatments, cells were washed twice with PBS, detached with a standard trypsinisation procedure and resuspended in PBS. The number of viable cells was determined by Trypan blue dye exclusion test.

### Cellular uptake

To assess the cellular uptake, Caco2 cells were grown up to confluence and were incubated for 24, 48 and 72 h with EC, EGCG, GA at 100 μM and IQ at 70 μM. The flavonoid content of cells was analysed by high-performance liquid chromatography (HPLC) equipped with a Coularray detector (ESA 5600) consisting of eight coulometric array cells. After treatment, cells were detached using a standard trypsinisation procedure, counted by haemocytometer, transferred to plastic tubes and washed in PBS. The cell suspension was washed three times with distilled water and subsequently lysed by sonication. Then, EGCG, EC, GA and IQ were hydrolysed and extracted twice with ethylacetate. The organic phase was evaporated under a stream of nitrogen and resuspended in HPLC mobile phase (methanol : water 1 : 1 v v^−1^) (Bugianesi *et al*, 2000). Aliquots (30 μl) were injected onto the HPLC; the mobile phase was methanol and water acidified at pH 2.8 with orthophosphoric acid. The elution gradient was 13–87% methanol in 38 min, with a flow rate of 1 ml min^−1^. The eight potentials were set from −80 to 200 for EGCG and EC and from 100 to 900 for IQ. The column used for separation was Supelcosil LC18, 25 cm in length and with an internal diameter of 2.1 mm (Supelco).

### Cell cycle analysis

In order to study the effect on the cell cycle, four cell cultures were incubated with 100 μM respectively of EC, EGCG, GA and 70 μM IQ for 24 h. Cell cycle distribution was assessed by determining BrdU incorporation *vs* DNA content. Cells were incubated with 30 μM BrdU during the last hour of culture, harvested and fixed in cold 70% ethanol. Fixed cells were washed in PBS, resuspended in 2 N HCl for 30 min at room temperature, pelleted and then resuspended in 0.1 N sodium tetraborate for 15 min. The samples were then washed in PBS, incubated for 15 min in PBS containing 1% bovine serum albumin and 0.2% Tween 20 (PBT) and for 30 min in 100 μl of anti-BrdU monoclonal antibody diluted 1 : 10 in PBT. After two washes with PBT, cells were incubated for 30 min with 100 μl of fluoresceinisothiocyanate (FITC)-conjugated anti-mouse antibody diluted 1 : 50 in PBT, then washed twice and resuspended in PBS containing 5 μg ml^−1^ propidium iodide (PI) and 100 μg ml^−1^ of RNAse A. Cells were analysed with a Coulter Epics XL (Coulter Corp.) flow cytometer. The instrument was equipped with an argon laser tuned at 488 nm for fluorescence excitation. Propidium iodide (PI) fluorescence was measured with a 585/44 or a 620/30 nm band-pass filter. Ten thousand cells were measured for each sample. Computer statistical analysis of mean fluorescence intensity (MFI) and graphic representation were performed with XL2 software (Coulter).

### Statistics

Statistical analyses were obtained by Anova test. For each analysis, at least three independent experiments were carried out. Data are given as the mean±s.d.

## RESULTS

### Antioxidant activity

TRAP values are shown in Table 1

**Table 1:**
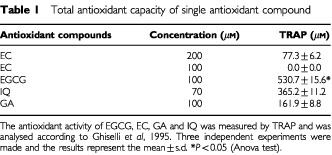
Total antioxidant capacity of single antioxidant compound

**Figure 1:**
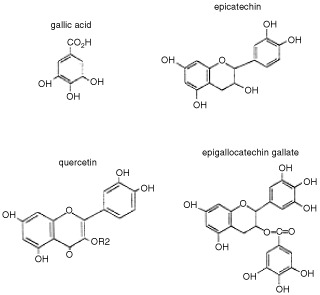
Chemical structure of flavonoids studied. The figure shows gallic acid, catechin, quercetin and epigallocatechin gallate. IQ: quercetin with R_2_=D-glucoside in the 3-position. EGCG differs from EC in having a galloyl moiety in the 3-position.

. Results are expressed as micromoles/l of peroxyl radicals scavenged by each antioxidant at the equimolar concentration (100 μM). Despite the similar chemical structure of flavonoids studied (Figure 1

), EGCG had the highest antioxidant activity against peroxyl radicals (530.7 μM, *P*<0.05) followed by IQ (365.2 μM, at 70 μM), GA (161.9 μM) and EC (0.0 μM). Since 100 μM EC did not show any detectable antioxidant activity, increasing concentrations (up to 200 μM) were also analysed. However, the TRAP value of 77.3 μM obtained at the highest EC concentration of 200 μM confirmed that this compound did not exert any antioxidant effect.

### Hydrogen peroxide production

Table 2

**Table 2:**
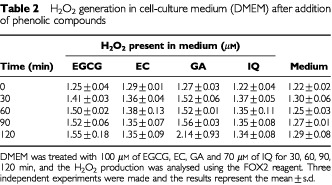
H_2_O_2_ generation in cell-culture medium (DMEM) after addition of phenolic compounds

shows the results obtained by the FOX2 assay. H_2_O_2_ generation was measured in the cell medium with and without addition of phenolic compounds. We observed insignificant production of H_2_O_2_ (<2 μM after 2 h) in presence of phenols with respect to control medium, under our experimental conditions.

### Cytotoxicity study

**Figure 2:**
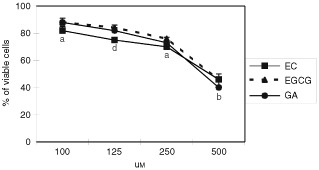
Cytotoxicity of selected flavonoids on Caco2 cell in culture. Cells were incubated with different concentrations of EGCG, EC, GA and IQ for 24 h and the number of viable cells was determined by Trypan blue test. Three independent experiments were made. Bars indicate standard deviation of the mean. a *vs* b *P*<0.05 (Anova test).

The threshold for toxicity was determined for each of the specified flavonoids. Figure 2

shows the toxicity, as evaluated by the Trypan blue test, for different concentrations of EC, EGCG and GA (100, 125, 250, 500 μM) in Caco2 cells after 24 h of incubation. The results are expressed as percentage of viable cells with respect to the survival of control cells. EC, EGCG and GA were found to be non toxic at the concentrations ranging between 100–250 μM with a cellular survival of 70–80%, whereas a 60% cytotoxicity occurred at 500 μM concentration of all flavonoids tested.

### Cellular uptake

Figure 3

**Figure 3:**
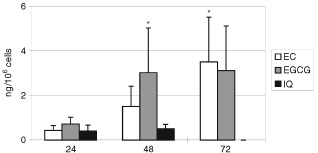
Time course of cellular uptake of EC, EGCG and IQ on Caco2 cells. Cells were incubated for 24, 48 and 72 h with 100 μM of EGCG, EC and with 70 μM of IQ, and the cellular uptake was analysed by HPLC. Three independent experiments were made. Bars indicate standard deviation of the mean. *P<0.05 (Anova test).

shows the time of course of cellular uptake of EC, EGCG, GA (100 μM) and IQ (70 μM) after 24, 48 and 72 h of incubation. Results are expressed as ng 10^6^ cells.

Epicatechin is taken up by Caco2 cells achieving levels of 0.43±0.22 ng/10^6^ cells after 24 h of incubation and reaching a value of 3.50±2.2 ng/10^6^ cells after 72 h of incubation. The final uptake of EGCG was comparable to that of EC (3.11±2.3 ng/10^6^ cells at 72 h) but reached this level earlier, at 48 h. In contrast, IQ was less readily absorbed compared to EC or EGCC, reaching a maximum of 0.50±0.20 ng/10^6^ cells after 48 h, and disappearing at 72 h.

GA was not detectable, both in the medium and in the cells, after 24, 48 and 72 h of incubation. This result was perplexing as GA exerts a clear effect on cell cycle parameters. Considering that it could be due to rapid degradation or binding of GA to a component of the medium, and/or a rapid metabolism, we measured the GA content both in the Caco2 cells and in the medium after 0, 10, 30, 60 min and 24 h of incubation. Table 3

**Table 3:**
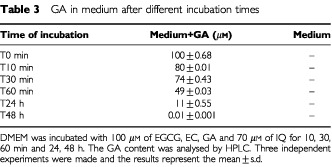
GA in medium after different incubation times

shows a 90% reduction of GA in the medium within 24 h of incubation. Similarly, Figure 4

**Figure 4:**
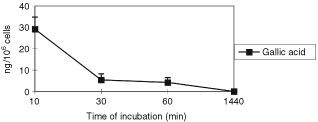
Time course of cellular uptake of GA on Caco2 cells. Cells were incubated with 100 μM of GA for 10, 30, 60 min and 24 h, and the cellular uptake was evaluated by HPLC. Three independent experiments were made. Bars indicate standard deviation of the mean.

shows that GA is readily taken up by cells and virtually disappears after 60 min (0.004 μg/10^6^ cells).

### Effect of flavonoids on cell cycle progression

**Figure 5:**
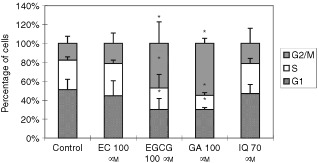
Effect of selected flavonoids on the cell cycle of Caco2 cells. Cells were incubated without or with 100 μM of EGCG, EC, GA and with 70 μM of IQ for 24 h, and the percentage of cells in each phase of the cell cycle was assessed by flow cytometry. Three independent experiments were made. Bars indicate standard deviation of the mean. *P<0.05 (Anova test).

The distribution of cells in each phase of the cell cycle after incubation for 24 h with 100 μM EC or EGCG or 70 μM of IQ, was determined using flow cytometry to quantitative DNA content (Figure 5

). Results are expressed as percentage of cells in each phase of cell cycle.

**Figure 6:**
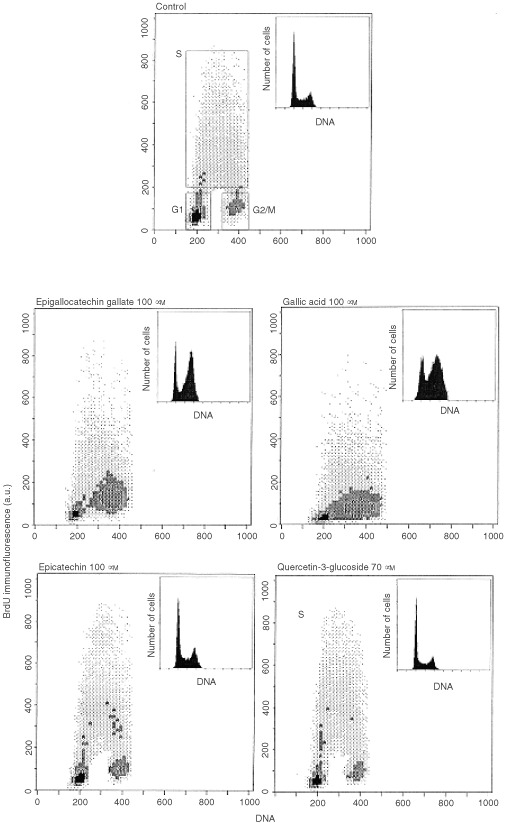
Frequency distributions of BrdU immunofluorescence *vs* DNA content in untreated and treated cells. Cells were incubated without and with 100 μM of EGCG, EC, GA and 70 μM of IQ for 24 h, and the percentage of cells in each phase was measured by flow cytometry. Three independent experiments were made.

EGCG and GA significantly increased the number of cells in G2/M phase (*P*<0.05), with a consequent reduction of cells in G1 and S phases. In fact, after 24 h of incubation, the number of cells in G2/M phase was increased by about 33 and 40% for EGCG and GA, respectively. On the contrary, responses to EC and IQ were similar to those of control cells. Figure 6

shows the dot plots of BrdU immunofluorescence *vs* DNA content in control Caco2 and in cells treated with EGCG, GA, EC or IQ for 24 h. The results show that cells treated with EGCG or GA incorporate significantly a lower amount of BrdU than control cells. Quantitative analysis of BrdU immunofluorescence, in the region corresponding to cells in S phase, indicated an inhibition in BrdU incorporation as a result of cells treated with the above flavonoids being delayed in G2/M phase.

## DISCUSSION AND CONCLUSION

A variety of micronutrients from plant products have been identified as compounds with potential anticarcinogenic properties. Among these micronutrients, phenolic compounds in food, and especially flavonoids, may exert a protective role against colon cancer. Although, there is much strong evidence to support this healthy effect of flavonoids in human and animal models, there is little knowledge about their bioavailability. The current flavonoid intake in Northern Europe has been estimated at around 50–150 mg day^−1^ for total flavonoids (Hollman and Arts, 2000). Generally less than 1% of the total amount ingested reaches the systemic circulation, and absorption commonly ranges from 0.2 to 0.5%. Most flavonoids are present in the diet as glycosides (Scalbert and Williamson, 2000), and quercetin and its glucosides have been the most extensively studied forms. Their absorption in man and in animal model is however a matter of controversy.

Although there are some studies of the absorption of EC and EGCG from green tea in human volunteers (Lee *et al*, 1995; Maiani *et al*, 1997) and animal models (Crespy *et al*, 1999) there are no reports on cellular absorption in the literature. This study assesses, for the first time, the uptake of the three free flavonoids EC, EGCG, GA and one flavonoid glucosides (IQ) in Caco2 cells. We observed a different uptake of each flavonoid studied in relation to their different chemical structure and to the position of glucose moiety. The EC content in cells steadily increased over 72 h of incubation, while cellular EGCG reached a maximum after 48 h of incubation. In the case of GA no detectable uptake by cells was observed after 24, 48 and 72 h. However, this was shown to be due to a rapid depletion of GA in the medium; 50% loss after 1 h and a maximum of 90% after 24 h. Cells were shown to rapidly take up GA within 10 min of the start of incubation. Considering the high reactivity of GA, it could be further metabolised or the metabolite bound to a medium component.

In the case of IQ we observed a rapid uptake by cells after 24 h of incubation, although its magnitude was lower than for the other three flavonoids. This is in agreement with hypotheses from two studies on the absorption of IQ in humans and in isolated rat intestine. Day *et al* (1998) demonstrated the presence of a cytosolic β-glucosidase in human small intestine having a high affinity for quercetin-4′-glucoside and genistein-7-glucoside but a low affinity for quercetin-3-glucoside. Furthermore, Gee *et al* (1998) showed an interaction between quercetin-3-glucoside and the sodium dependent glucose transporter (SGLT1) in rat intestine. Walgren *et al* (1998), working with cultures of Caco2 cells, evaluated the transport of quercetin and its glucosides, mainly quercetin-4′-glucoside, quercetin-3,4′-diglucoside. These authors showed that transport depended on the position and nature of glucosidic moiety and hypothesised that a glucose moiety in the 3-position could promote absorption and vice versa with respect to glycosylation at the 4′-position. Recently, the same authors (Walgren *et al*, 2000a,b) have demonstrated for the first time that quercetin-4′-glucoside is transported by SGLT1 across the apical membrane of enterocytes. However, its trancellular absorption is limited by a multidrug resistance-associated protein MRP2 mediated efflux across the apical membrane as well as by an unknown transporter on the basolateral membrane. Despite a direct evidence about the transport of quercetin-4′-glucoside (Gee *et al*, 1998; Walgren *et al*, 2000a,b) there are some controversial results that require further research.

A number of investigators have reported that flavonoids inhibit the tumour growth by interfering with some phases of the cell cycle. Green tea extract and quercetin have been the most extensively studied agents. Quercetin shows anti-proliferative effects against various cancer cell lines (Yoshida *et al*, 1990), markedly inhibiting the proliferation of gastric and colon cancer cells and arresting the cell cycle at G1 phase. Moreover, quercetin was found to inhibit the cell growth of leukaemia T-cells (Yoshida *et al*, 1992), Ehrlich ascite tumour cells (Suolinna *et al*, 1975) and NK/Ly ascite tumour cells. Agullo *et al* (1994) have demonstrated that quercetin has potent cytotoxicity against colon cancer cells *in vitro*, and that this is associated with antiproliferative activity. Literature data (Khafif *et al*, 1998) show a synergistic chemopreventive effect between EGCG and curcumin on the cell cycle of premalignant and malignant human oral epithelial cells. Flow cytometric studies have showed that EGCG and curcumin inhibited growth by different mechanisms; EGCG blocked all cells in the G_1_ phase, whereas curcumin blocked cells in S/G_2_M phases. Another study (Suganuma *et al*, 1999) has showed a synergistic effect between EC and EGCG on cell growth inhibition and on inhibition of TNF-α release, an endogenous tumour promoter of human lung cancer cell line PC-9. Ahmad and Mukhtar (1999) have reported that EGCG induced apoptosis and cell cycle arrest in human carcinoma cells. These results suggest that green tea polyphenols, particularly EGCG, can act as antitumour, promoters, antiproliferators, be anti-inflammatory, and may be useful for cancer chemoprevention (Liang *et al*, 1999).

Our study shows that EGCG and GA induce a cell cycle delay in Caco2 cells. Treatments with 100 μM EGCG and GA increased the percentage of cells in G_2_/M phase by 33 and 40% respectively, whilst 100 μm EC and 70 μM quercetin-3-glucoside, which do not contain a galloyl moiety did not inhibit the progression of the cell cycle. We also observed a decrease in G_1_-phase cell associated with an accumulation in G_2_/M phase. These data confirm the findings of Okabe *et al* (1997) in human lung cancer cell line PC9 incubated with the same concentration of EGCG.

A link between cell cycle arrest and antioxidant capacity has been postulated for EGCG and GA. We have also evaluated the ability of EGCG and GA to scavenge free radicals (measured as TRAP). The relative order of decreasing efficiency was EGCG>IQ>GA>EC suggesting a possible correlation for EGCG but not for GA. Thus, the apparent antioxidant activity of flavonoids does not relate to the inhibition of cell growth as also observed by Kuntz *et al* (1999).

Another possible mechanism by which the anticarcinogenic activity of the flavonoids may be mediated, could be the induction of apoptosis. We therefore determined the frequency of apoptosis by monitoring the typical morphological changes of nuclear chromatin distribution after staining cells with Hoechtst 33258 dye. Caco2 cells incubated with flavonoids showed a similar pattern to control cells. Thus the flavonoids tested failed to induce apoptosis in our model system (data not shown).

Recently, Long *et al* (2000) have demonstrated that the addition of GA, EGCG, EC, quercetin and catechin to commonly used cell culture media leads to generation of substantial amounts of hydrogen peroxide (H_2_O_2_). The authors suggested that H_2_O_2_ production by these phenolic compounds could be responsible of their effect on cells. Particularly, the gallate moiety seems especially important, since GA and EGCG generated substantial amounts of H_2_O_2_. However, we have measured the H_2_O_2_ production in our samples and no significant generation of H_2_O_2_ was observed, comparable results being obtained with and without addition of phenolic compounds.

In conclusion, the results of this study indicate that, among dietary flavonoids studied, EGCG or GA are able to interfere with the cell cycle in a Caco2 cell line, and that EGCG, IQ and GA show a significant antioxidant effect in a chemical model system. From a structural point of view, the galloyl moiety appears to be required both for the antioxidant and antiproliferative effects.

The fact that the intestinal epithelium can be exposed to high concentration of dietary flavonoids, implies that these compounds could have an important role in the prevention of colon cancer through the inhibiting the cell cycle of epithelial cells. However, the *in vitro* inhibition of growth of cancer cell line does not necessarily infer down regulation of growth of the entire epithelium *in vivo*.

Moreover, the protective effect of fruit and vegetables as a whole could be greater than any one single phenolic compound, probably due to the presence of many antioxidants that could, together, potentiate this effect.
